# Curiosity and Mind Wandering During Music Listening Are Negatively Correlated

**DOI:** 10.3390/bs15030393

**Published:** 2025-03-20

**Authors:** Juan Felipe Pérez Ariza, Diana Omigie

**Affiliations:** 1 Department of Cognition, Emotion, and Methods in Psychology, University of Vienna, 1010 Vienna, Austria; jpere004@gold.ac.uk; 2 Department of Psychology, Goldsmiths, University of London, London SE14 6NW, UK

**Keywords:** music, mind wandering, curiosity, musical expertise, information theory, learning

## Abstract

Curiosity, a crucial trigger of exploration and learning, has been described as the antithesis of mind wandering, a state of non-engagement with the external environment or a given task. Findings have confirmed that music’s structure influences levels of curiosity in listeners as they listen and, as such, suggests that this context could be useful in examining the relationship between curiosity and mind wandering. Here, participants were exposed to extended melodies twice, during which they carried out two counterbalanced tasks: one requiring them, whenever probed, to indicate whether they had been mind wandering at that moment and the other requiring them to indicate, when probed, how curious they were feeling about the music at that moment. Critically, participants were probed at the exact same moments in the music when completing the two tasks, allowing the relationship between curiosity and mind wandering to be examined. Results confirmed our prediction of a negative relationship between curiosity and mind wandering, while exploratory analysis further suggested an influence of expertise and the music’s information dynamics on patterns of mind wandering. We discuss the implications of our study for understanding music as an exploration-affording sound environment and outline directions for future work.

## 1. Introduction

Recent theory conceptualises curiosity as a form of motivated cognition that, by evoking information-seeking behaviours, favors knowledge acquisition in everyday life (Gottlieb et al., 2013; Scacco & Muddiman, 2020; van Lieshout et al., 2020). Defined as a momentary desire for knowledge (Loewenstein, 1994), curiosity is repeatedly linked to reward processes in the brain (Cervera et al., 2020; Gruber et al., 2014; Kidd & Hayden, 2015; Murayama et al., 2019; Sakaki et al., 2018). However, while curiosity is recognised as a core cognitive state underlying patterns of attentional engagement and exploratory behaviours in a wide range of everyday contexts (Hassan et al., 2015; Ligneul et al., 2018), the domains in which it has been empirically explored remain limited.

Two recent studies have demonstrated the relevance of using music, a highly ubiquitous stimulus, to understand how curiosity is influenced by the structure of the environment. Specifically, through a continuous rating paradigm and time-course analysis approach, Omigie and Ricci (2022) showed that music-induced feelings of curiosity can be explained by listeners’ perception of change in the music. Extending that work, another study from the same authors showed that the information content (surprisingness) and entropy (unpredictability) of unfolding musical events were reflected in listeners’ self-reports of curiosity while listening (Omigie & Ricci, 2023). Importantly, these studies build on a large body of work that demonstrates that listeners are constantly making predictions about how music will unfold and that proposes that these (music expectancy processes) constitute the basis of how music evokes meaning and emotion in music (Huron, 2006; Meyer, 1956). However, they extend that work by providing empirical evidence that attentional dynamics during extended music listening episodes can be at least partly explained by the information dynamics of the unfolding music (Omigie & Mencke, 2023). They also complement existing models of attentional dynamics in music, like the dynamic attending theory, which focuses on how rhythmic structure, in particular, influences internal attentional oscillations (Jones, 1976; Bauer et al., 2015).

The recognition that levels of attention directed towards a piece of music may vary over the course of the listening experience (with peaks in attention being interspersed with moments of mind wandering (Omigie & Mencke, 2023) resonates with the Region of Proximal Learning (RPL) model of curiosity (Metcalfe et al., 2020), which claims that curiosity and mind wandering are two components of the same information seeking process. The first iteration of the RPL model (Metcalfe, 2002) aimed to account for how learners focus their resources, whether it be study time allocation or attention. Concretely, according to the original RPL hypothesis, sensing that they are on the verge of knowing or understanding a stimulus induces individuals to enter their RPL zone, a state of persistent engagement in which learning and engaging with a stimulus is not only easier but also more enjoyable. The RPL model of curiosity (Metcalfe et al., 2020) describes state curiosity as arising from increases in the learner’s metacognitive feeling that there is a potential for learning in the environment or task. Critically, it predicts that, when not experiencing this state of curiosity (because the stimulus to which they are exposed does not match their abilities, being either too easy or too difficult for them to learn), individuals will tend to mind wander.

The RPL model of curiosity is motivated by similar previous work (Berlyne, 1954, 1966), which suggested that people show epistemic curiosity for things that are at the right level of complexity. The RPL also builds upon the ideas of Piaget (1954) and others, who suggest that curiosity tends to occur when individuals perceive that they “almost-know” the answer to a query regarding a stimulus. However, the RPL model of curiosity’s core prediction of a negative correlation between curiosity and mind wandering remains to be systematically examined. Similarly, while mind wandering, defined as a shift in attention away from the external environment or current task to an internally oriented flow of thoughts and images (Christoff et al., 2016; Seli et al., 2018), is increasingly studied in music psychology (Taruffi, 2021), the factors underlying its dynamics remain under-researched.

The present study aimed to both (i) test the RPL hypothesis of a negative relationship between instances of curiosity and mind wandering (main hypotheses) using music as a testing ground, as well as (ii) advance understanding of how listeners engage with music over extended timescales. Exposing participants to musical stimuli over extended listening episodes, we used the probe-caught paradigm to assess the occurrence of mind wandering and listeners’ degree of curiosity about the unfolding music at the exact same musical moment across two repetitions of the heard music. Critically, we predicted that moments in a piece of music where a given listener reported high levels of curiosity would constitute moments (in another listening episode) where they would also show a reduced likelihood of mind wandering. Assessing listener expertise, varying the complexity of the musical stimuli, and estimating its information dynamics allowed further exploration of how these might influence patterns of mind wandering (exploratory analyses). Here, we hypothesised that as has been shown with curiosity (Omigie & Ricci, 2023), dynamics of mind wandering may be partly explainable by listeners’ level of expertise and the music’s global and local stimulus properties.

## 2. Methods

### 2.1. Participants

The experimental procedure was approved by the University of Goldsmiths’ Ethics Committee. To estimate a minimum sample size with which to find a discernible effect for our main hypothesis, we simulated synthetic datasets with a range of sample sizes and analysed them with the model that was most plausible to have a good out-of-sample performance (see Supplementary Materials S2); a rationale that has been used to estimate sample size elsewhere (Gelman & Hill, 2007; McElreath, 2020). As it was seen that the likelihood of finding a medium-size effect was 80% with 20 participants, 26 individuals (16 Females; age: M = 28.36, SD = 9.2) were recruited, with all participants receiving monetary compensation. Participants’ scores on the Music Training subscale of the Goldsmiths Musical Sophistication Index (Müllensiefen et al., 2014), which, when averaged, produces a value between 1 and 7, showed a good distribution of sophistication levels around the average (M = 4.04, SD = 1.83). To illustrate using two items of the scale, 30.5% of participants reported having never had formal music theory training, while 38.5% of participants reported having never had formal training in voice or a musical instrument.

### 2.2. Stimuli

All participants were presented with two unfamiliar melody sets that differed only in how much unpredictability they presented an enculturated Western listener. One melody set comprised three melodic sequences, separated by a space of 3640 ms, each of which had been composed according to Western tonal rules using the open-source music notation software MuseScore version 4.40. Each melodic sequence in this “original melodies set” comprised 80 bars of exclusively quarter notes (thus 320 notes per melodic sequence), where changes to a related key occurred every 16 bars (thus 5 key changes in total).

The second melody set was created from the “original melodies set” described above by randomising the order of notes within each of the three melodic sequences using the “choice” function of the “random” module in Python’s (version 3.13.2) standard library. This manipulation reduced the structural predictability of each of the melodic sequences in the “shuffled melodies set” while keeping the structure of the set (three melodies separated by a space of 3640 ms) and each melodic sequence’s ingredients (e.g., constituent pitches) the same.

The increased unpredictability of melodies in the “shuffled melodies set” was confirmed by the entropy values obtained using a computational model of melodic expectations (Pearce, 2018), employed in a configuration commonly used in the literature (Hedges & Wiggins, 2016; Omigie & Ricci, 2023; Pearce, 2005; Pearce et al., 2010). In the field of information theory, entropy can be defined as the average level of information or uncertainty that is inherent in a variable’s possible outcomes. Critically, in line with early studies using music theoretic approaches to characterise stimuli (Besson & Faïta, 1995; Koelsch & Rohrmeier, 2012), musical events characterised as high in entropy are associated with feelings of uncertainty (Hansen & Pearce, 2014; Hansen et al., 2016). Accordingly, and as can be seen in Table 1, entropy values were consistently higher in the shuffled melodic sequences than in the original melodic sequences. Supplementary Materials S1 includes entropy distributions for both types of stimuli and the pitch distribution for all stimuli. A comparison of original (M = 2.75, SD = 0.2) and shuffled melodies (M = 3.2, SD = 0.02) showed a significant difference (M = −0.46, SD = 0.02, 89% PI [−0.5, −0.42]) in entropy.

Finally, to increase overall levels of engagement with the task of listening to what were largely simple melodic sequences, the tempo and timbre of the sequences were manipulated in a systematic way in the two melodic sets. Critically, while no hypotheses were posed around how levels of curiosity and rates of mind wandering may change as a function of tempo and timbre (these being two fairly arbitrary features that, as such, were not analysed), we assumed that having the melodic sequences switching between fast and slow tempos and different timbres would drive some of the variations in curiosity and mind wandering that would allow the nature of their relationship to be examined in this study. Table 1 summarises these stimulus details. With regard to tempo, the first and third melodies in each set were rendered faster than the second melody. Specifically, in these two fast-paced (first and last) melodies in the set, each note had a duration of 455 ms, and the duration of each melody was thus 2 min and 25 s. In contrast, in the slow-paced melody (second) in each set, notes had a duration of 910 ms, and the duration of the melody was 4 min and 50 s. With regard to timbre, the first melody in both sets was rendered in Violin, the second in French Horn, and the third in Vibraphone. All stimuli (and data scripts) can be found in the following project repository: https://osf.io/7f9jm/?view_only=0f799edaf94d40099f0127f020eab3c2 (accessed on 10 February 2025).

### 2.3. Procedure

Stimuli and data were presented and collected using the graphical experiment builder OpenSesame (Mathôt et al., 2012). Participants were presented with the auditory stimuli using around-ear Behringer headphones, and their responses were recorded using a keyboard and a mouse on a PC running Windows 10.

**Figure 1:**
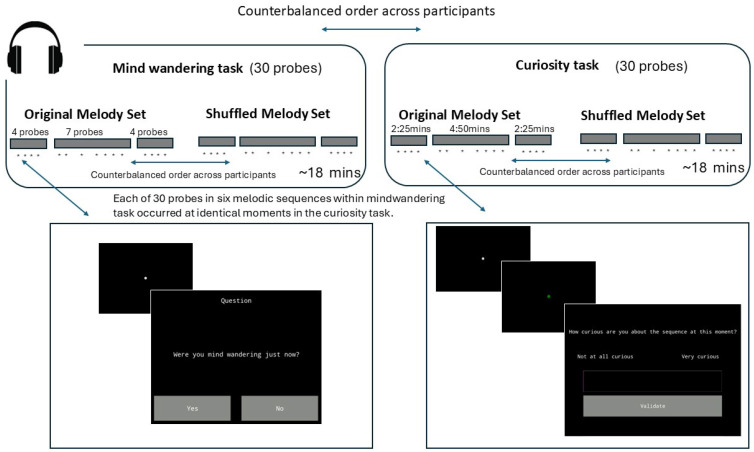
Schematics of the experimental protocol. Participants carried out a curiosity and mind wandering task, the order of which was counterbalanced across participants. The timing of the sequence of prompts was generated individually for each participant and was identical both times the participants heard each melody. The question for the mind wandering task prompt read, “Were you mind wandering just now?” with the options “yes” and “no” presented. The question for the curiosity task prompt read, “How curious are you about the sequence at this moment?” with the left extreme of the slider being “Not at all curious” and the right extreme “Very curious”.

Figure 1 shows a detailed description of the procedure, depicting the experience of an example participant. Participants performed two tasks (a mind wandering task and a curiosity task), the order of which was counterbalanced. For each of these tasks, participants were presented with both the Original melodies set (original set of three melodic sequences) and the Shuffled melodies set (three melodic sequences created by shuffling the notes within each of the original set of three melodic sequences). Similar to what was implemented for the order of the curiosity and mind wandering tasks, the order in which participants were presented with the original and shuffled melodic stimuli was also counterbalanced across participants, although the order of the music type was kept the same across both tasks within a participant (i.e., both times the original and then the shuffled melody sets, or vice versa).

For both tasks, participants were probed regularly in intervals ranging from 30 to 45 s while they listened to the sequences, although the first probe they experienced was displayed at a random interval between 0 and 30 s. Critically, the probe for a given melody for a given task occurred at the exact same moment when the melody was presented again for the alternative task. This allowed examination of how an individual participant’s mind wandering report at a given point in a melody was predicted by their curiosity level at the exact same moment. A unique sequence of intervals (including the interval between the beginning of the sequences and the first probe) was generated for each participant to enhance the confidence with which generalisations from the study can be made.

In each task, participants experienced 15 probes while listening to each melody set: 4 from each of the two fast sequences and 7 from the slow ones. In the mind wandering task, participants indicated whenever probed whether they were mind wandering. Participants were told that “mind wandering is a state in which thoughts drift away from the material being presented. When you are mind wandering, your thoughts may drift to memories of past events, friends, or even concerns about an upcoming exam”. In the mind wandering task, participants were asked to focus on a white fixation dot in the middle of the screen until a probe appeared at which point they were required to answer the question “Were you mind wandering just now?” with either the button “yes” or “no”. In the curiosity task, participants were required to focus on a white fixation dot on the screen, similar to the mind wandering task. However, whenever a probe was about to appear on the screen, the white fixation dot changed its colour and increased in size over a short period (1820 ms or 3640 ms for fast and slow melodies, respectively) to prepare the listener for the response slider that consequently appeared. Participants could then indicate their answer to the question “How curious are you about the sequence at this moment?” using the slider from the extremes of “Not at all curious” to “Very curious”. In both tasks, the probes appeared, and participants provided their answers without the music being interrupted at any point.

Each probe was shown on the screen while 6 notes of the current melodic sequence were playing (thus 2730 ms for fast and 5460 ms for slow melodies). Although participants were told to answer as fast as they could when they saw the probes, the question remained on the screen for as long as it was scheduled to do so. Providing a lead-up to the curiosity probe, but not the mind wandering task, was implemented to ensure that the moments of music-listening responses that were being compared across the two tasks maximally overlapped. Specifically, by allowing participants to start evaluating their experience in the lead-up to the curiosity probe, we could be more certain we were matching the music-driven experience of curiosity to the music-driven experience of mind wandering (which, by definition, would have to have started at least a short period before the probe for an affirmative answer to be accurate). Critically, we did not include a lead-up to the mind wandering task since that, by definition, would take participants out of the state of mind wandering as well as render the task too different from how it tends to be implemented. Importantly, while participants’ curiosity and mind wandering were probed in slightly different ways, this difference did not impact our ability to test our hypothesis of a relationship between curiosity and mind wandering.

Both tasks were preceded by a short practice opportunity that allowed participants to familiarise themselves with the task. Participants were asked after the mind wandering task to answer two multiple-choice questions (about the order in which they had heard the instruments playing) and to indicate whether or not brief excerpts presented to them had been in the melodies. As this was performed to corroborate instructions for the mind wandering task, which emphasised attentive listening, the data were not used as a variable for analysis.

Finally, participants completed the Music Training subscale of the Goldsmiths Musical Sophistication Index (Müllensiefen et al., 2014) at the end of the last task. This allowed us to explore how the musical training of participants might influence their patterns of mind wandering.

### 2.4. Analysis

Data were analysed using R version 4.4.0 (R Foundation for Statistical Computing, 2024), fitting the models to Stan with the rethinking package (McElreath, 2020; Stan Development Team, 2022).

We hypothesised that mind wandering would be negatively related to curiosity and modelled this using multilevel logistic regression. The curiosity ratings variable, with a continuous range from 0 to 1, was used to predict mind wandering occurrence, a dichotomous variable. To find the best-performing model, Pareto-smoothed importance sampling (PSIS) cross-validation scores were computed and compared, along with their out-of-sample standard error (i.e., the standard error of their mean accuracy at predicting new data) and weights. PSIS scores offer an approximate gauge of out-of-sample deviance of models and thus allow estimation of which one will perform better with future data, with lower values being better (Vehtari et al., 2017). The Hamiltonian Monte Carlo algorithm was used to sample from the joint posterior distributions of each parameter assessed in the models, obtained by Bayesian updating. Finally, models were fitted with regularising priors, allowing partial pooling to update estimates across clusters and inform those with few observations.

## 3. Results

Models were fitted incrementally such that, initially, only the fixed effect of curiosity on mind wandering was included, while later models included varying intercepts for participants and melodic sequences and varying slopes for sets of melodic stimuli (original and shuffled). Supplementary Materials S3 contains the comparison between all the models fitted and shows that models with varying intercepts for melodic stimuli and uncertainty conditions and varying slopes for melodic stimulus did not result in notable differences. Accordingly, a model that only included varying intercepts for participants was used to make inferences.

**Figure 2:**
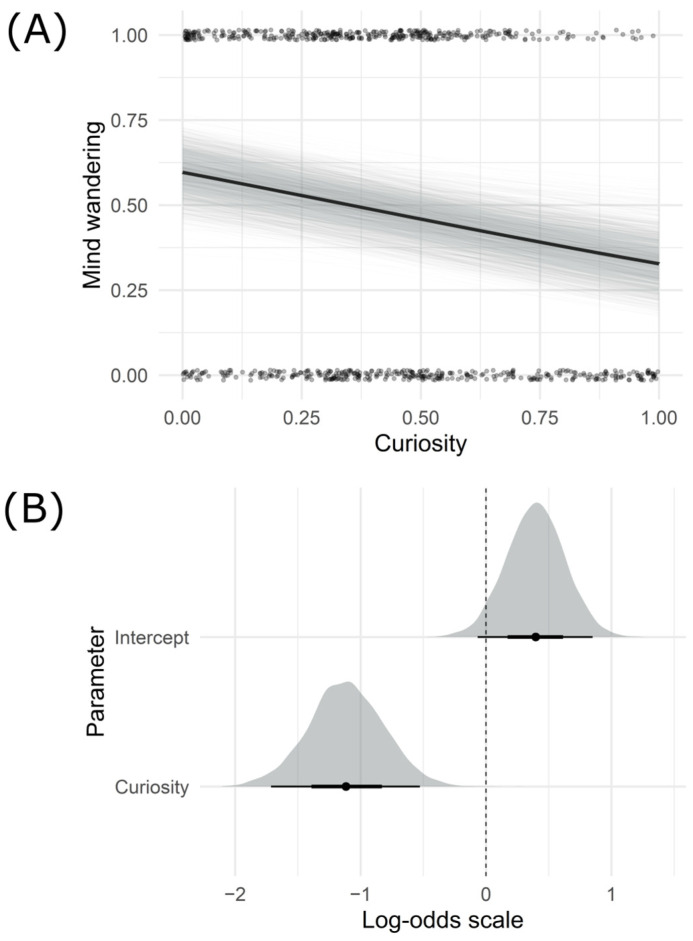
Average tendency between curiosity and mind wandering and posterior distributions of curiosity and intercept for the best model. (**A**) The dark black line reflects the average tendency between mind wandering and curiosity. Light grey lines are drawn from the joint posterior distribution, and translucent black points represent curiosity ratings. (**B**) Posterior distributions (posterior median and 95% PI) were estimated for the parameters. Black points represent the median of the distribution. Thick black lines represent one standard deviation. Thin black lines represent two standard deviations. An estimate is thought to have a meaningful inference when most of its density lies above or below 0.

This resulting model showed a negative relationship between mind wandering and curiosity (β = −1.12, SD = 0.30, 89% Percentile Compatibility Interval (PI) = [−1.61, −0.64]). Specifically, it showed that, for every positive change in curiosity rating, participants were 28% less likely to mind wander. Importantly, the fact that the 89% PI does not contain 0 indicates that this effect is unambiguously negative in our dataset. The average effect of curiosity on mind wandering, plotted against the raw data, is displayed in Figure 2, along with the posterior distributions for the curiosity variable.

Having provided support for our main hypothesis, we carried out exploratory analyses to examine whether mind wandering occurrence was influenced by listener expertise and stimulus type (original versus shuffled melodies, where the latter was higher in global entropy as estimated using IDyOM). We also explored whether mind wandering at a given moment could be predicted from the local uncertainty/entropy (also as estimated using IDyOM) of the music at that moment. In both cases, we split our sample according to participants’ score on the Music Training subscale of the Goldsmiths Musical Sophistication Index, which, when averaged, produces a value between 1 and 7; we considered participants with a score higher than 4 as having high expertise (M = 5.50, SD = 0.96), and those with a score lower than 4 as having low expertise (M = 2.30, SD = 0.82).

Regarding whether mind wandering occurrence was influenced by stimulus type and expertise, a multilevel logistic regression in which stimulus type (original versus shuffled) was allowed to interact with expertise was fitted. While the fact that at least 18% of its posterior density lies above 0 suggests that this was not the case for all participants, results suggested that expertise had a small negative effect on mind wandering, reducing the likelihood by 2% (β = −0.08, SD = 0.09, 89% PI = [−0.23, 0.07]). In contrast, neither the stimulus type (original versus shuffled) nor its interaction with expertise seemed to have any effect on mind wandering (Original Stimuli: β = 0.08, SD = 0.37, 89% PI = [−0.51, 0.68]; Shuffled Stimuli: β = 0.03, SD = 0.37, 89% PI = [−0.56, 0.62]; Original Stimuli—Expertise: β = 0.05, SD = 0.29, 89% PI = [−0.52, 0.42]; Shuffled Stimuli—Expertise: β = −0.03, SD = 0.29, 89% PI = [−0.49, 0.43]).

Regarding whether mind wandering could be predicted from the momentary entropy of the sequence (and therefore the uncertainty listener might be experiencing at that moment) and whether any such effect was influenced by listener expertise, exploratory multilevel logistic regression analyses showed that momentary entropy increased mind wandering likelihood by 5% (Entropy: β = 0.20, SD = 0.20, 89% PI = [−0.11, 0.51]), that participants with lower musical expertise were 11% more likely to mind wander than those with higher musical expertise (β = −0.11, SD = 0.1, 89% PI = [0.05, −0.27]) and that while participants with high musical expertise tended to mind wander less as entropy values increased, the opposite was true for those with low expertise (Low Expertise—Entropy: β = 0.10, SD = 0.31, 89% PI = [−0.39, 0.58]; High Expertise—Entropy: β = −0.10, SD = 0.30, 89% PI = [−0.59, 0.38]). However, the fact that the posterior distributions for the exploratory findings did not lie exclusively at one side of 0 reflects the variability of the results in our sample and the tentative character of these results.

## 4. Discussion

Recognition that levels of attention directed towards a piece of music may vary over the course of the listening experience, with peaks in attention being interspersed with moments of mind wandering (Omigie & Mencke, 2023), resonates with a key claim of the RPL model of curiosity (Metcalfe et al., 2020), namely that curiosity and mind wandering are two components of the same information seeking process. A key claim of the hypothesis is that curiosity is negatively correlated with mind wandering, a state of disengagement from a task at hand. The current study used music as a testing ground for evaluating this claim and showed that curiosity was indeed negatively correlated with mind wandering. Exploratory analyses also suggested that higher expertise was associated with lower mind wandering and that mind wandering reflected the information dynamics of the musical stimuli.

A growing body of work has begun to explore mind wandering in the context of music listening, showing that it is just as prevalent during music listening as in other sustained tasks (Deil et al., 2023; Taruffi, 2021). However, that work has tended to focus on the rate of occurrence and the content and emotional qualities of these moments of mind wandering. Our finding that curiosity during music listening is negatively correlated with mind wandering can be seen as an important extension and corroboration of both that work and other research examining how feelings of curiosity change as a function of music’s information dynamics (Omigie & Ricci, 2023). Notably, previous studies have relied on subjective self-report of curiosity alone, a measure that may be subject to demand characteristics on the part of the participants, whereby they may report feeling curious when they perceive the music as more surprising, regardless of any accompanying desire to actually keep engaging. That the current study was able to show a relationship between self-reports of curiosity and mind wandering, a behaviour proposed to be negatively correlated with curiosity, corroborates previous findings and highlights the relevance of using music to study both curiosity and mind wandering and the relationship between the two.

Our exploratory analyses also suggested that with increasing expertise, listeners may become less susceptible to mind wandering at complex music moments, a finding in line with the RPL, where engagement with difficult and complex instances of stimuli have been shown to be easier as exposure and mastery increase (Metcalfe, 2002). These findings are also in line with a large body of music psychology research showing that musicians tend to respond quite differently from naïve listeners when exposed to musical stimuli (Baumann et al., 2008; Darrow et al., 2006; Farbood et al., 2015; Penhune, 2019), including in response to conspicuously complex musical stimuli (Mencke et al., 2019, 2022). Indeed, the current study supports the notion that the dynamics of attentional engagement with a piece of music are influenced by listener characteristics, amongst other factors (Omigie & Mencke, 2023).

Taken together, our findings support the idea of music in the environment as affording opportunities for engagement and exploration (Reybrouck et al., 2024). Future work could use the current paradigm to ask a range of questions about both the music-listening experience and the dynamics of attentional engagement more broadly. For example, it is relevant to ask whether the relationships found here would hold in more complex and naturalistic everyday listening scenarios. Here, it is interesting to consider recent work that tries to explain how deep engagement with a piece of music can seem to involve close attention to sonic features alongside what seems like mind wandering (Høffding et al., 2024); this by emphasising how attention in music can be argued to go beyond a focus on acoustic features to encompass imagination, affect and emotional intentionality. In our study, the melodies were unfamiliar and largely lacking in emotional depth, and mind wandering was described to listeners with examples that emphasised thoughts unrelated to the heard music. Future work, in which real musical pieces with higher familiarity are used as stimuli, will provide a useful test of the extent to which concepts like curiosity and mind wandering can be considered distinct or mutually exclusive in the kinds of emotion-rich and imagination-affording contexts that music listening affords.

In any case, it is worth considering the promise of future research that recognises curiosity as an intrinsic part of the music listening process. As previously noted, while curiosity has been widely examined in connection with learning and exploratory behaviours, the psychology of music has only rarely addressed it to date. With this study, we hope to have shown the potential of using the concept of curiosity as a valuable point of departure in any research that seeks to explore the dynamics of music listening.

## 5. Conclusions

In sum, while epistemic emotions are recognised to play an important role in music listening (Huron, 2006; Meyer, 1956), the use of music to explore the proposed dichotomy between curiosity-driven attentional engagement and mind wandering is a strategy that has not yet been fully exploited. The observation that mind wandering and curiosity are negatively related during music listening is not only useful in providing support for the RPL hypothesis but also provides fresh insights into the temporal dynamics of attentive music engagement.

## Figures and Tables

**Table 1:** Description of Stimuli.

Melodic Set	Melodic Sequence	Entropy (M(SD))	Timbre	Time Signature	Number of Bars	Number of Pitches	Pitch Duration (ms)	Total Duration (minutes)	Key Changes
Original melodies (low entropy)	1	2.70 (0.53)	Violin	4/4	80	320	455	2:25	5
	2	2.65 (0.52)	French Horn	4/4	80	320	910	4:50	5
	3	2.88 (0.53)	Vibraphone	4/4	80	320	455	2:25	5
Shuffled melodies (high entropy)	1	3.13 (0.56)	Violin	4/4	80	320	455	2:25	NA
	2	3.19 (0.53)	French Horn	4/4	80	320	910	4:50	NA
	3	3.30 (0.55)	Vibraphone	4/4	80	320	455	2:25	NA

Note. ms = Milliseconds.

## Data Availability

The original data presented in the study are openly available in the following Open Science Framework repository: https://osf.io/7f9jm/?view_only=0f799edaf94d40099f0127f020eab3c2 (accessed on 10 February 2025).

## Supplementary Materials

The following supporting information can be downloaded at https://www.mdpi.com/article/10.3390/bs15030393/s1, Supplementary Materials S1: Pitch and Entropy Distributions for Type of Stimulus. Supplementary Materials S2: Sample Size Calculation. Supplementary Materials S3: Model Comparison for Main Hypothesis.
