# Supplementary material for: Curiosity and Mind Wandering During Music Listening Are Negatively Correlated
Source: Behav Sci (Basel). 2025 Mar 20;15(3):393. doi: 10.3390/bs15030393 (PMC11939264; doi:10.3390/bs15030393)
Supplement: Supplementary file 1 [file behavsci-15-00393-s001.zip › behavsci-3497103-supplementary.pdf]

S1 – Pitch and Entropy Distributions for Type of Stimulus

Figure S1.1

Distribution of Pitches in stimuli

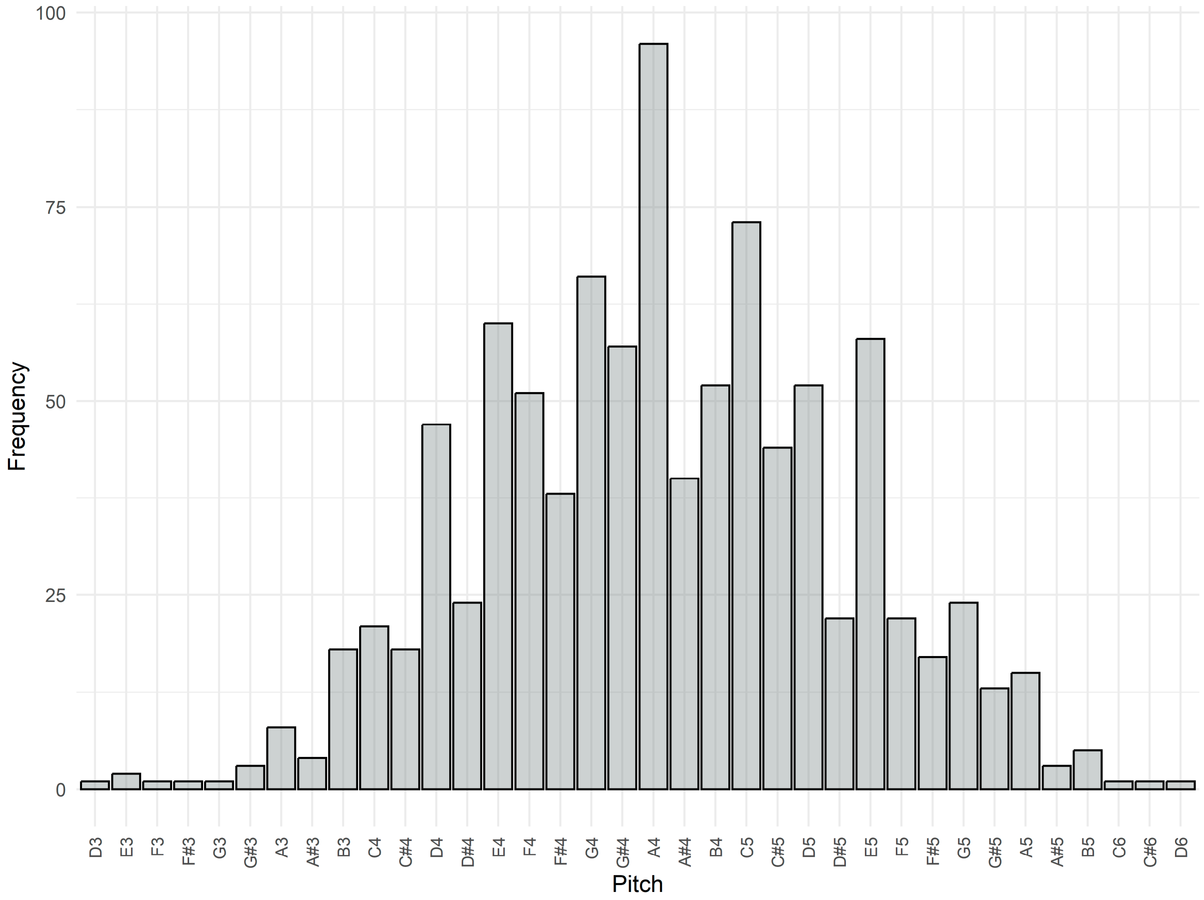

Figure S1.2

Entropy Distributions for Original and Shuffled Stimuli

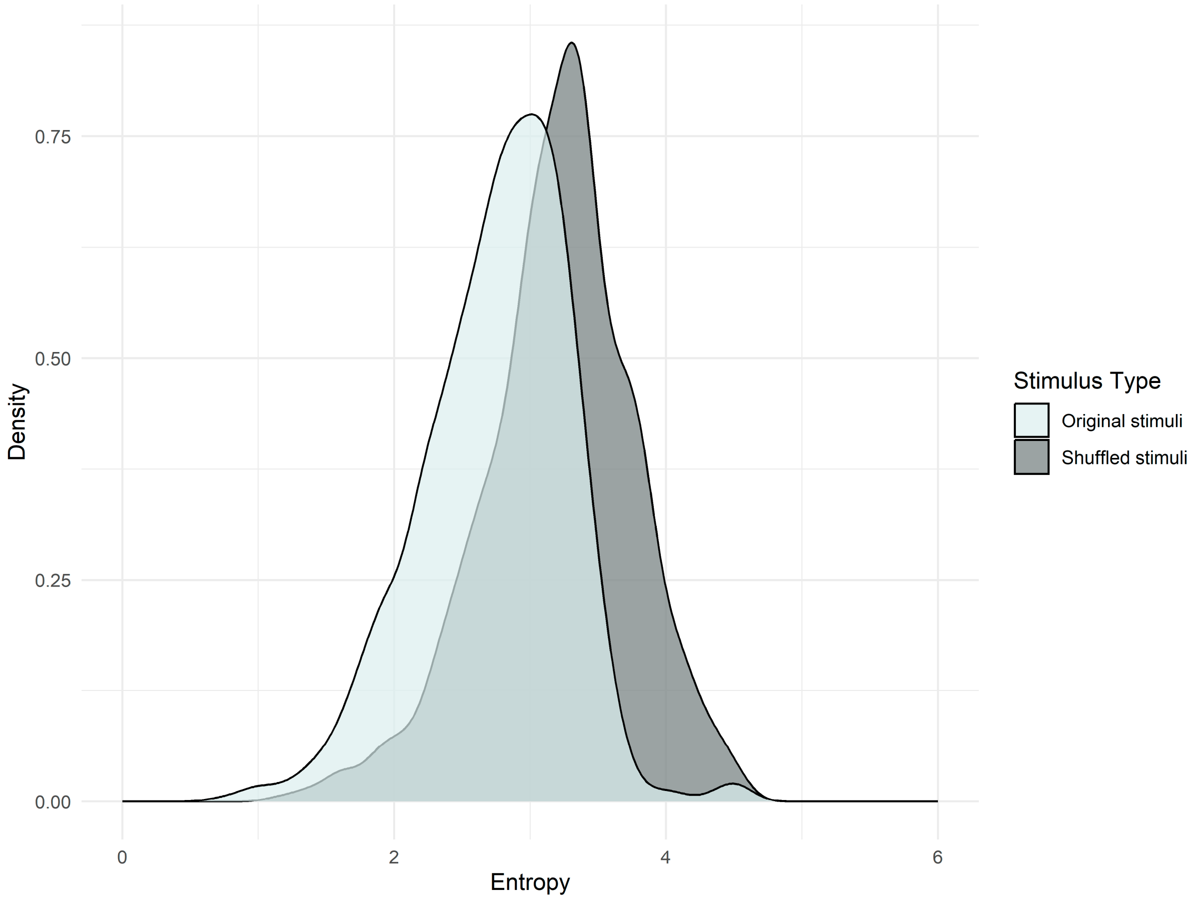

S2 – Sample Size Calculation

To estimate a minimum sample size with which we would be able to find a discernible effect, we simulated 500 synthetic datasets with a range of sample sizes and analyzed them with a model we thought would plausibly describe our data well.

Thus, for each sample size, we generated a synthetic dataset and then fitted the model to these data. We repeated this process 5 times per sample size, and we assessed the proportion of model fits in which the effect of curiosity on mind-wandering was estimated to be two standard deviations below 0. The model that we used allowed intercepts to vary for each participant, as we expected to find considerable individual variation in response to the musical stimuli.

We simulated the data with the following assumptions.

Initially, since our main hypothesis did not stratify by stimulus type, we hypothesized that the likelihood of mind-wandering among participants in response to the stimuli, in general, was of 50%. This implies that we expected participants to be equally likely to mind-wander, in average, irrespective of the type of stimulus condition to which they were listening. Then, to estimate a medium effect size, we hypothesized that curiosity would decrease participants' likelihood of mind-wandering in, at most, 12.5%. This change in probability would be estimated to be a coefficient of -0.5 in the log-odds scale.

It is worth mentioning that ours is a repeated measures design. Specifically, the experimental design takes 60 observations per participant: 15 per variable (mind-wandering frequency and curiosity ratings) and block, and, as discussed in the main text, participants completed two blocks, one for each task.

The power curve that results from the simulations can be seen in Figure 1. As it can be observed, the likelihood to find a medium size effect was of 80% with, at least, 20 participants. This suggests that our sample size was sufficient to assess the main hypothesis of the study.

Figure S2.1

Sample size calculation based on simulations

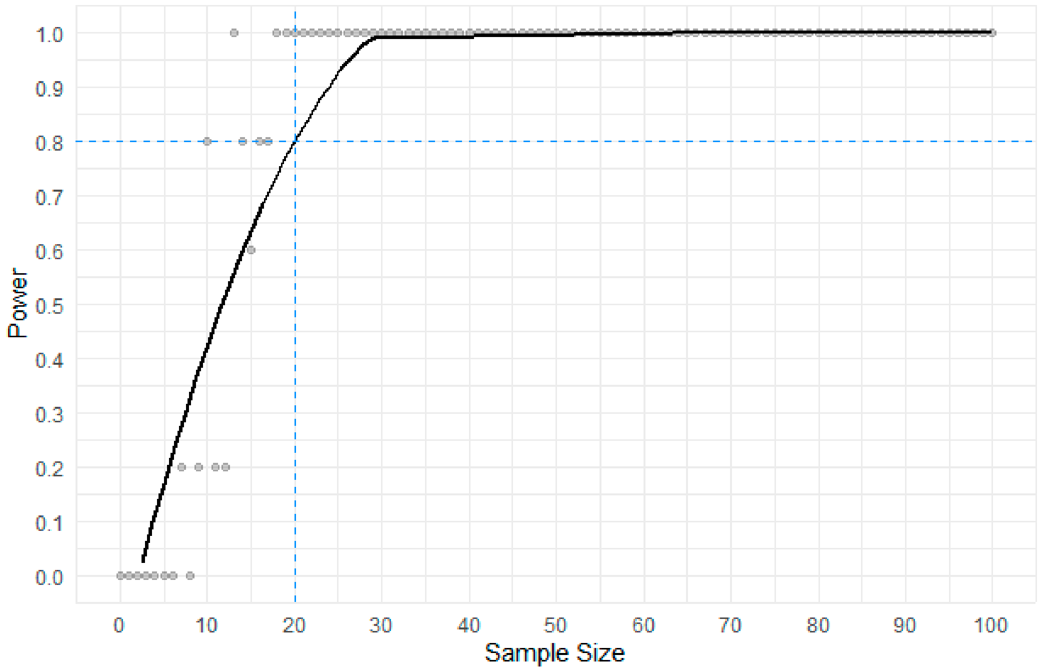

Note. Each point represents the likelihood that the estimate of the effect of curiosity over mind-wandering lies two standard deviations below 0, using the corresponding sample size. Dashed lines connect a probability of 80% on the Y axis with the corresponding sample size in the X axis on the line of best fit.

S3 – Model Comparison for Main Hypothesis

Table S3.1 displays the comparison between the models. The model that includes varying intercepts for participants performs better out of sample. However, the standard error of the difference between the three top models estimated by the comparison implies that none of them are easily distinguishable. This suggests that any of them would perform similarly in predicting new data, and as such, using any of them to make inferences would not result in considerably different estimations. To emphasize this similarity, Table 2 includes the main parameters (curiosity and mindwandering) estimated by each model.

Table S3.1

Model comparison for the main hypothesis

	PSIS Score	SE	PSIS Difference	SE of the Difference	PSIS Penalty	Weight
Model 1	853.12	19	0	0	22.18	.48
Model 2	854.06	20.04	.94	2.26	24.79	.30
Model 3	854.06	20.08	.94	2.30	24.87	.21
Model 4	860.87	20.12	7.75	4.99	34.33	.01
Model 5	961.68	10.65	108.56	19.16	1.79	0
Model 6	1000	.75	146.88	19.87	1	0

Table S3.2

Comparison of parameters estimated by the three top models to evaluate the main hypothesis

Varying Effects	Parameter	M	SD	PI 5.5%	PI 94.5%	R hat	Eff. Samples
VI: Participant	Model 1						
	Intercept	.39	.24	.01	.77	1	1777.59
	Curiosity	-1.11	.30	-1.60	-.64	1	5873.71
VI: Participant & Melodic Stimuli	Model 2						
	Intercept	.35	.25	-.07	.73	1	2888.13
	Curiosity	-1.07	.30	-1.56	-.59	1	8324.85

VI: Participant & Melodic Stimuli	Model 3						
	Intercept	.28	.33	-.26	.77	1	2361.28
	Curiosity	-1.09	.31	-1.58	-.59	1	5722.09

Note. VI = Varying Intercepts. VS = Varying slopes. PI = Percentile Compatibility Interval

As such, considering that the models do not estimate much variation neither in individual melodic stimuli nor in type of stimulus (see Table S3.3), we used the model that only includes varying intercepts for participants to make inferences.

Table S3.3

Variation estimated by the top models to evaluate the main hypothesis

Cluster of Variation	M	SD	PI 5.5%	PI 94.5%	1	Eff. Samples
Model 1						
Participant	1.12	.21	.83	1.49	1	2124.19
Model 2						
Participant	1.12	.21	.83	1.49	1	2392.16
Melodic Stimuli	.20	.16	.02	.48	1	2910.21
Model 3						
Participant	1.13	.22	.83	1.51	1	1774.27
Melodic Stimuli	.37	.43	.02	1.16	1	2461.37
Type of Stimulus	.23	.19	.03	.54	1	2603.84

Note. PI = Percentile Compatibility Interval

Thus, the model used to make inferences can be summarized as follows:

$$\text{Mind Wandering} \sim \text{Binomial}(1,\ p_i)$$

$$\text{Logit}(p_i)\ = \overline{\alpha} + z_{Participant[i]}\sigma_{\alpha} + \beta_{\text{Curiosity}}\ \text{Curiosity}_i$$

$$\beta_{\text{Curiosity}} \sim \text{Normal}(0, 0.5)$$

$$z_i \sim \text{Normal}(0, 1)$$

$$\overline{\alpha} \sim \text{Normal}(0.\ 0.5)$$

$$\sigma_{\alpha} \sim \text{Exponential}(1)$$

As mentioned in the main text, we used regularizing priors on the possible effect of curiosity over mindwandering. Thus, by providing its effect a normal distribution with mean 0 and standard deviation of 0.5, the model would be less likely to consider extreme values as highly plausible but attribute the same likelihood to the possibility of the effect being positive or negative.

Equally important, we used a non-centred prior on the varying intercepts for participants to avoid common sampling problems associated with posterior distributions with steep areas. Although our sample size included a good number of clusters (participants) to estimate variation among participants, we found that the centred versions of the models resulted in a few hundred of non-divergent transitions, and so, we decided to fit the models in this manner.
